# Efficient and Reliable Production of Vectors for the Study of the Repair, Mutagenesis, and Phenotypic Consequences of Defined DNA Damage Lesions in Mammalian Cells

**DOI:** 10.1371/journal.pone.0158581

**Published:** 2016-06-30

**Authors:** Lucy Petrova, Christine Gran, Magnar Bjoras, Paul W. Doetsch

**Affiliations:** 1 Program in Genetics and Molecular Biology, Emory University, Atlanta, Georgia, United States of America; 2 Department of Biochemistry, Emory University School of Medicine, Atlanta, Georgia, United States of America; 3 Department of Microbiology, Oslo University Hospital and University of Oslo, Oslo, Norway; 4 Department of Cancer Research and Molecular Medicine, Norwegian University of Science and Technology, Trondheim, Norway; 5 Department of Radiation Oncology, Emory University School of Medicine, Atlanta, Georgia, United States of America; 6 Department of Hematology and Medical Oncology, Emory University School of Medicine, Atlanta, Georgia, United States of America; 7 Winship Cancer Institute, Emory University School of Medicine, Atlanta, Georgia, United States of America; University of Massachusetts Medical School, UNITED STATES

## Abstract

Mammalian cells are constantly and unavoidably exposed to DNA damage from endogenous and exogenous sources, frequently to the detriment of genomic integrity and biological function. Cells acquire a large number of chemically diverse lesions per day, and each can have a different genetic fate and biological consequences. However, our knowledge of how and when specific lesions are repaired or how they may compromise the fidelity of DNA replication or transcription and lead to deleterious biological endpoints in mammalian cells is limited. Studying individual lesions requires technically challenging approaches for the targeted introduction of defined lesions into relevant DNA sequences of interest. Here, we present a systematic analysis of factors influencing yield and an improved, efficient and reliable protocol for the production of mammalian expression phagemid vectors containing defined DNA base modifications in any sequence position of either complementary DNA strand. We applied our improved protocol to study the transcriptional mutagenesis-mediated phenotypic consequences of the common oxidative lesion 5-hydroxyuracil, placed in the G12 mutational hotspot of the KRAS oncogene. 5-OHU induced sustained oncogenic signaling in Neil1^-/-^Neil2^-/-^ mouse cells. The resulting advance in technology will have broad applicability for investigation of single lesion DNA repair, mutagenesis, and DNA damage responses in mammalian cells.

## Introduction

Mammalian cells are continuously exposed to DNA damage, which can be detrimental to health and is associated with cancer, neurodegenerative disease and aging [1–3]. Exposure to a variety of unavoidable endogenous sources, such as cellular respiration, and ubiquitous exogenous sources, such as UV light, X-rays and chemical agents, can result in a vast array of known and unknown lesions [4–6]. At the molecular level, DNA lesions can cause variable levels of DNA or RNA polymerase stalling or mutagenic bypass during replication or transcription [7]. Similarly to DNA polymerase, when RNA polymerase encounters DNA lesions, it can misincorporate incorrect nucleotides opposite to the lesions resulting in transcriptional mutagenesis (TM). At the cellular level, DNA lesions can induce senescence or cell death [8], and at the organismal level they can lead to disease [3]. The consequences of DNA damage have been extensively studied in systems where populations of cells are exposed to DNA damaging agents. Such treatments result in, often extensive, DNA damage in the form of a variety of known and unknown lesions, at random genomic positions unique for each cell. Much can be understood regarding DNA damage and repair using these systems. However, delineating the fates and consequences of each specific lesion, in physiological contexts with or without the potentially confounding factor of additional damage that can induce phenotypes related to the levels of damage rather than any individual lesion, requires a more targeted approach. DNA and RNA polymerase bypass efficiencies, lesion stability and replicative or transcriptional mutagenicity, as well as the type of mutation introduced when mutagenesis occurs differ for each specific lesion, and different lesions are recognized and repaired by different components of the DNA repair pathways. All of these factors, as well as the specific sequence position in which a lesion occurs, can determine biological outcomes. Each particular lesion, and the unique context in which it occurs, could differentially contribute to human disease. For example, a highly mutagenic lesion occurring in an oncogene mutational hotspot and evading DNA repair is likely to result in detrimental biological consequences, in contrast to one that is rarely mutagenic or quickly repaired.

DNA repair of defined lesions can be studied *in vitro* using radiolabeled synthetic oligodeoxynucleotides (ODNs) containing the desired modification, however this precludes the study of any potential phenotypic consequences, and biochemical characterizations may not be reproducible *in vivo*. For example, in a reconstituted *in vitro* transcription system, the common oxidative lesion 8-oxoguanine (8-oxoG) hinders transcription, nonetheless in HeLa nuclear extracts [9] and *in vivo* in bacterial [10] and mammalian [11, 12] cells it is a miscoding lesion, efficiently bypassed by RNA polymerase, inducing transcriptional mutagenesis and phenotypic change. However, the ability of other oxidative lesions, such as 5-hydroxyuracil (5-OHU) or dihydrouracil (DHU) to compromise the fidelity of transcription *in vivo* and induce phenotypic change have not been investigated. If left unrepaired, they have the potential to induce more pervasive and long-lasting phenotypic change due to significantly higher levels of mutagenesis following RNA polymerase (RNAP) bypass that have been demonstrated *in vitro* [9, 13].

Several methods, with different advantages and limitations, have been developed for the study of individual lesions *in vivo*. One strategy, the gapped duplex method, involves the digestion of a plasmid using sequence-specific nicking enzymes and exchanging the excised ODN with one containing the lesion of interest [14]. However, the applications of this approach are limited, as it requires the presence of two tandem nicking endonuclease recognition sequences adjacent to the lesion site. A more versatile approach involves the annealing of a synthetic lesion-containing ODN to a single-stranded vector followed by enzymatic complementary strand synthesis. While the single-stranded (ss) M13 phage genome can also be used [15], the use of ss phagemid vectors [16, 17] permits the use of any mammalian expression vector, containing any feature and gene or sequence of interest, so long as it also contains the f1 phage origin of replication. Such mammalian expression vectors containing site-specific base modifications in any position or sequence of interest are an enabling technology for the study of the consequences of defined DNA damage lesions occurring in targeted positions of protein-coding regions of genes, such as oncogene mutational hotspots, in mammalian cells [12]. Also, when the lesion of interest is strategically placed in a reporter gene, such as a fluorescent protein from which the normal fluorescent sequence is transcribed only when transcriptional mutagenesis occurs, these vectors allow for the study of the mechanisms and regulation of DNA damage repair as well as monitor repair capacities and repair dynamics of known lesions of interest in live mammalian cells [18]. The use of these tools complements and can vastly extend our understanding of DNA damage and repair in contexts relevant to human health. However, their construction poses significant technical challenges. The necessary protocols are complex, time-consuming and laborious, they can require the use of expensive or toxic chemicals, and can result in low or unpredictable yields. Mammalian transfection requires large quantities of highly pure DNA, however the factors that determine yield and protocol reproducibility are not well characterized.

In order to simplify the protocol and determine factors influencing yield, reproducibility, and achieve highest final product quality, we performed systematic analyses of the steps for the production and purification of single-stranded phagemid DNA and double-stranded vectors containing site-specific DNA damage lesions for applications in mammalian cell culture systems. We present an optimized protocol yielding large quantities of ultra-pure, double-stranded, lesion-containing vectors well suited for mammalian transfection. In order to improve protocol reproducibility between different preparations and experimenters, we provide reliable predictors of yield. We applied this improved protocol to study the phenotypic consequences resulting from mutagenic transcriptional bypass of 5-OHU placed the G12 mutational hotspot of KRAS, such that when TM occurs due to the misincorporation of adenine opposite to 5-OHU, it would result in the production of constitutively active KRAS^G12D^ transcripts and proteins activating downstream effectors of Ras. Incorporation of guanine opposite this cytosine-derived lesion, due to it not being transcriptionally mutagenic or repaired, would result in wild type transcripts and proteins. We find that in Neil1^-/-^Neil2^-/-^ (Nei endonuclease VIII-like 1 and 2) DNA glycosylase deficient mouse embryonic fibroblasts, 5-OHU induces sustained transcriptional mutagenesis-mediated oncogene activation, implicating Neil1 and/or Neil2 in the repair of 5-OHU *in vivo*. Such oncogene activation via a TM mechanism, in comparison to that mediated by 8-oxoG shown previously [12], is sustained much longer than previously known. Moreover, we show TM activates AKT–a pathway downstream of Ras previously not known to be activated via a TM mechanism. Thus, the continuous time course of transcriptional mutagenesis-mediated changes in cellular signaling and the activation of multiple downstream effectors of Ras can potentially be significant through inducing a variety of detrimental physiological consequences.

## Materials and Methods

### Plasmids and Cloning Procedures

The backbone for all vectors used was pcDNA3.1(+) (Invitrogen). pcDNA3.1(+) vectors contain the f1 origin of replication in the forward direction, such that the non-transcribed strand is the produced ssDNA. EGFP was sub-cloned from pEGFP-N1 into pcDNA3.1(+) by restriction digestion using BamHI and NotI and standard cloning procedures. The HRAS^WT^ and KRAS^WT^ plasmids containing the human sequences were from Guthrie cDNA Resource Center. The HRAS^Q61K^ plasmid has been previously described [12]. The KRAS^G12D^ mutant was produced using the KRAS^WT^ plasmid and the QuickChange Site-Directed Mutagenesis Kit (Agilent Technologies) according to the manufacturer’s instructions and primers with the sequence CTCTTGCCTACGCCATCAGCTCCAACTACC (forward) and GGTAGTTGGAGCTGATGGCGTAGGCAAGAG (reverse).

### *Escherichia coli* Culture and M13KO7 Phage

DH12S *E*. *coli* cells (Invitrogen Cat. #18312–017), which are endA+ in order to mimimize dsDNA production, were transformed with pcDNA3.2(+) plasmids containing the insert of interest and grown in LB-Miller medium containing 100 μg/mL carbenicillin at 37°C and 225 rpm. On the day of phage infection, cultures were diluted 1:500 in 2X-YT medium with carbenicillin. For large-scale cultures, 200 mL of culture in 2 L baffled flasks were used, and for small-scale cultures, used for systematic analysis of phage infection conditions, 20 mL in 250 mL baffled flasks. Cells were infected with M13KO7 phage stock at > 1 x 10^11^ pfu/mL (Invitrogen Cat. #18311–019) at the MOI indicated for each sample. The *E*. *coli* density was determined by measuring OD_600_ and assuming OD_600_ of 1.0 = 8 x 10^8^ cells/mL. The cultures were then incubated for 30 minutes without shaking and an additional 1.5 hours with shaking before adding kanamycin to a final concentration of 75 μg/mL and incubating with shaking overnight. The next day, bacteria were pelleted by centrifugation and supernatants filtered through a low protein binding 0.22 μm filter (Corning, Cat. #431097).

### SDS Phage Lysis and Anion-Exchange ssDNA Purification

PEG-8000 was purchased from Sigma. Phage were precipitated by the addition of 0.2 volumes solution M1 (3 M NaCl and 30% (w/v) PEG-8000) and incubating at 4°C for ≥ 1 hr. Phage were pelleted at ≥ 10,000 x g for 20 minutes at 4°C, supernatants decanted and pellets drip-dried for approximately 5 minutes. The pellets from one to two 200 mL starting cultures were purified using a single midi column from the PureLink® HiPure Plasmid Midiprep Kit (Invitrogen, Cat. # K2100-04). Briefly, each pellet was resuspended in 3 mL buffer M2 (100 mM Tris-HCl, pH 8.0 and 25 mM EDTA), then 3 mL solution M3 (4% SDS) was added, samples were mixed by inversion and then incubated at 70°C for 20 min. Then, 3 mL buffer N3 were added, samples were again mixed by inversion, and centrifuged at 14,000 x g for 10 min at room temperature. The supernatants were applied to columns pre-equilibrated with buffer EQ1 and DNA purified according to the manufacturer’s instructions, except that the elution buffer (E4) was pre-warmed to 50°C before use. Pellets were resuspended in TE buffer, yields quantified using NanoDrop spectrophotometry, and a small sample of each ssDNA visualized on a 0.7% agarose gel containing ethidium bromide to ensure purity.

For the silica spin column purifications, phage were precipitated and ssDNA purified using QIAprep spin M13 kit (Qiagen, Cat. #27704) according to the manufacturer’s instructions and samples were pooled.

### Determination of Phage Yield by Proteinase K Digestion

In order to determine amounts of ssDNA, buffer M1 precipitated phage were pelleted at 14,000 x g on a table-top microcentrifuge at 4°C for 20 min. Pellets from each mL of culture were resuspended in 50 μL TE pH 8.0 containing 100 μg proteinase K (Invitrogen, Cat. #25530–015) and 0.1% SDS and incubated at 42°C for 1 hr. The samples were then resolved on 0.7% agarose TBE gels containing ethidium bromide. Phagemid ssDNA band intensities were quantified using the ImageQuant TL software.

### Oligodeoxynucleotide Annealing

PAGE-purified, 5’ phosphorylated lesion-free and 8-oxoguanine-containing ODNs, with sequences as indicated in S1 Table, were purchased from Eurofins MWG Operon, and those containing 5-hydroxyuracil or dihydrouracil were purchased from Midland Certified Reagent Company. 80 pmole of ODN, from 100 μM stock in TE buffer, were added for every 10 μg of ssDNA and annealed in 1X saline-sodium citrate (SSC) buffer, in a final volume of 50 μL, at 75°C for 10 minutes in a sterile microcentrifuge tube placed in a water beaker in a 37°C water bath, after which they were allowed to cool slowly to room temperature. Amicon Ultra-0.5 30K centrifugal filter units (Millipore, Cat. # UFC503024), which are recommended for efficient removal of primers ranging from 10–48 bases, were used according to the manufacturer’s instructions to concentrate the products and remove salts and unannealed ODNs. Briefly, for each sample, PCR grade water was added to a final volume of 500 μL, and then the concentration centrifugation step was carried out at 14,000 x g for 10 minutes and the elution step at 1,000 x g for 2 minutes.

### Second Strand Synthesis

PEG-8000 was dissolved in nuclease-free water and filtered through a 0.45 μm filter. T4 DNA ligase (Cat. #15224–017) and T4 DNA polymerase (Cat. #18005–017) were from Invitrogen. All other reagents were purchased from New England Biolabs. Second strand synthesis was performed overnight as previously described [16], scaling up or down as necessary. Polymerization reactions starting with 80 μg of ssDNA annealed with ODN were carried out in 1X NEBuffer 2 (10 mM Tris-HCl, 10 mM MgCl2, 50 mM NaCl, 1 mM DTT, pH 7.9), containing 7.5% PEG-8000, 50 μg/mL BSA, 600 μM each dNTP, 1 mM ATP, 80 U T4 DNA ligase, and 40 U T4 DNA polymerase, in a total volume of 600 μL. The samples were incubated for 5 min on ice, then 5 min at room temperature, before incubating at 37°C overnight. In order to enzymatically digest nicked, linear and single-stranded DNA, T5 exonuclease (New England Biolabs, Cat. #M0363) was added for the samples indicated in the text, directly to the second strand synthesis reaction or to purified dsDNA product in buffer NEBuffer 2 at 5 units per μg of starting ssDNA or dsDNA, respectively, and incubated for an hour at 37°C. Reactions were stopped by the addition of EDTA to a final concentration of 11 μM and DNA purified.

### dsDNA Purification Using Anion-Exchange Columns

Double-stranded products were purified using anion-exchange column kits (Qiagen, Cat. #12123 and 12243), using the products of no more than 150 μL second-strand synthesis reaction per Tip-20 column with capacity of 20 μg and 600 μL per Tip-100 column. Each sample was resuspended in at least 10 volumes of 750 mM NaCl, 50 mM MOPS pH 7.0 resuspension buffer, then applied to a Tip-20 or Tip-100 column pre-equilibrated with buffer QBT. The remainder of the protocol was according to the manufacturer’s instructions for plasmid mini- or midiprep procedures. Purified DNA pellets were resuspended in buffer TE, pH 8.0 and yields were determined using NanoDrop spectrophotometry.

### Fpg and Nth Nicking Assays

250 ng of each construct were digested with Formamidopyrimidine DNA glycosylase (Fpg, New England Biolabs, Cat. #M0240S) using 1 μL of Fpg (8 units) in the presence of BSA, according to the manufacturer’s instructions, in 1X NEBuffer 1 for 1 hr at 37°C. Products were separated and visualized on 0.7% agarose TBE gels containing ethidium bromide. For the Endonuclease III (Nth) assay, Endonuclease III (New England Biolabs, Cat. # M0268S) in 1X Endonuclease III reaction buffer was used instead.

### Alkaline Gel Electrophoresis

T5 exonuclease treated or Fpg nicked control constructs were purified using a PureLink® PCR Purfication kit (Invitrogen, Cat. #K3100-01) as per the manufacturer’s instructions, and eluted in nuclease-free dH_2_O. All restriction enzymes were obtained from New England Biolabs. Constructs were digested with SmaI for 90 min at 25°C in 1X CutSmart® Buffer, NdeI was added and the samples were incubated at 37°C for 90 minutes, and then enzymes were heat inactivated at 65°C for 20 min. Samples were ethanol-precipitated and resuspended in 1X alkaline gel loading buffer, then heated for 10 minutes at 75°C, and cooled on ice for 3 minutes before loading on 0.6% alkaline gels. Alkaline agarose gel electrophoresis was performed as previously described [19]. After neutralization, gels were stained four times, 15 minutes each, with 0.5 μg/mL ethidium bromide in TAE buffer and destained in TAE buffer without ethidium bromide.

### Cell Culture and Mammalian Transfection

Primary mouse embryonic fibroblasts (MEFs) were generated from 13.5 days old C57BL/6 Neil1^-/-^/2^-/-^ embryos (Gran *et al*, manuscript in preparation). Limbs were removed from embryos, the tissue was chopped into small pieces and cell suspension was made by pipetting vigorously. MEFs were grown in DMEM medium (Gibco) supplemented with 10% fetal bovine serum (FBS, Sigma), 2 mM glutamine (GlutaMAX, Gibco) and 1x penicillin/streptomycin (Gibco). Cells grown for 4–5 days were frozen at passage 2 in DMEM medium with 20% FBS/10% DMSO. Neil1^-/-^Neil2^-/-^ MEFs displayed the same proliferation rate as wild type MEFs. Experimental procedures were approved by the Norwegian Animal Research Authority. MEFs were immortalized by frequent passaging, using the 3T3 protocol as described previously [20]. Neil1^-/-^Neil2^-/-^ and Ogg1^-/-^ MEFs, described previously [12], were cultured in a humidified incubator at 10% CO_2_ in high-glucose DMEM containing GlutaMAX (Invitrogen) supplemented with 10% fetal bovine serum (GE Healthcare). MEFs were electroporated using an Amaxa Nucleofector 2B device and MEF 1 Nucleofector® kit (Lonza, Cat. #VPD-1004) using the T-020 setting as per the manufacturer’s instructions and 3–4 μg of DNA per 1.0 x 10^6^ to 1.5 x 10^6^ cells.

### Western Blot Analysis

Cells were washed with PBS, and switched to serum-free DMEM 1.5 hours before lysis. Samples were lysed using RIPA (150 mM NaCl, 1% Triton X-100, 0.5% sodium deoxycholate, 0.1% SDS, 50 mM Tris pH 8.0) freshly supplemented with cOmplete protease inhibitor cocktail tablets (Roche, Cat. #04693159001) and Halt phosphatase inhibitor cocktail (ThermoFisher Scientific, Cat. #78420). Ten to twenty μg of protein were resolved using 10% NuPAGE Bis-Tris gels (Life Technologies) and transferred onto PVDF membranes for two hours at 80V. Antibodies against phospho-AKT (Ser473, Cat. #4060), AKT (pan, Cat. #4691), phospho-ERK1/2 (Thr202/Tyr204, Cat. #9106) and ERK (Cat. #9102) were from Cell Signaling Technology, and K-Ras was from Santa Cruz Biotechnology (Cat.# sc-30). Membranes were blocked for 1 hour at room temperature in 2% ECL Prime blocking reagent (GE Healthcare, Cat. # RPN418) diluted in TBST, and antibodies were diluted 1:3,000 (pAKT and AKT), 1:200 (pERK1/2 and ERK), or 1:500 (K-Ras) in blocking buffer. Secondary horseradish peroxidase-conjugated antibodies were from Promega and diluted 1:5,000 (anti-mouse) or 1:10,000 (anti-rabbit) in blocking buffer. Three washes in TBST were carried out after each antibody incubation, and the membranes were developed for ECL and exposed to film. Membranes were cut and blotted for pAKT or pERK and K-Ras, then stripped and re-probed for total AKT or ERK. Film was scanned, images quantified using ImageQuant TL, the ratio of (pAKT/AKT)/K-Ras was determined for each sample and expressed as percent of the mutant positive control which was set to 100%.

Composition of buffers and media not described in Materials and Methods can be found in S2 Table.

## Results

### Reliable Predictors of Single-Stranded Phagemid Yield

In order to produce phagemid single-stranded DNA (ssDNA), we infected log-phase DH12S *E*. *coli* cultures with the M13KO7 derivative of the M13 phage that preferentially packages phagemid ssDNA containing the f1 origin of replication, rather than its own genome, which it packages in the absence of phagemid [21]. Which specific complementary strand of the phagemid is replicated and packaged depends on the orientation of the f1 origin. For the study of transcriptional mutagenesis, we employed a vector, pcDNA3.1(+), containing f1 in the forward direction and for which the non-transcribed strand undergoes single stranded replication, allowing the annealing of a lesion-containing ODN which would become part of the transcribed strand. For investigations of lesions on the non-transcribed strand, a vector containing f1 in the opposite orientation, such as pcDNA3.1(-), can be used instead.

**Fig 1:**
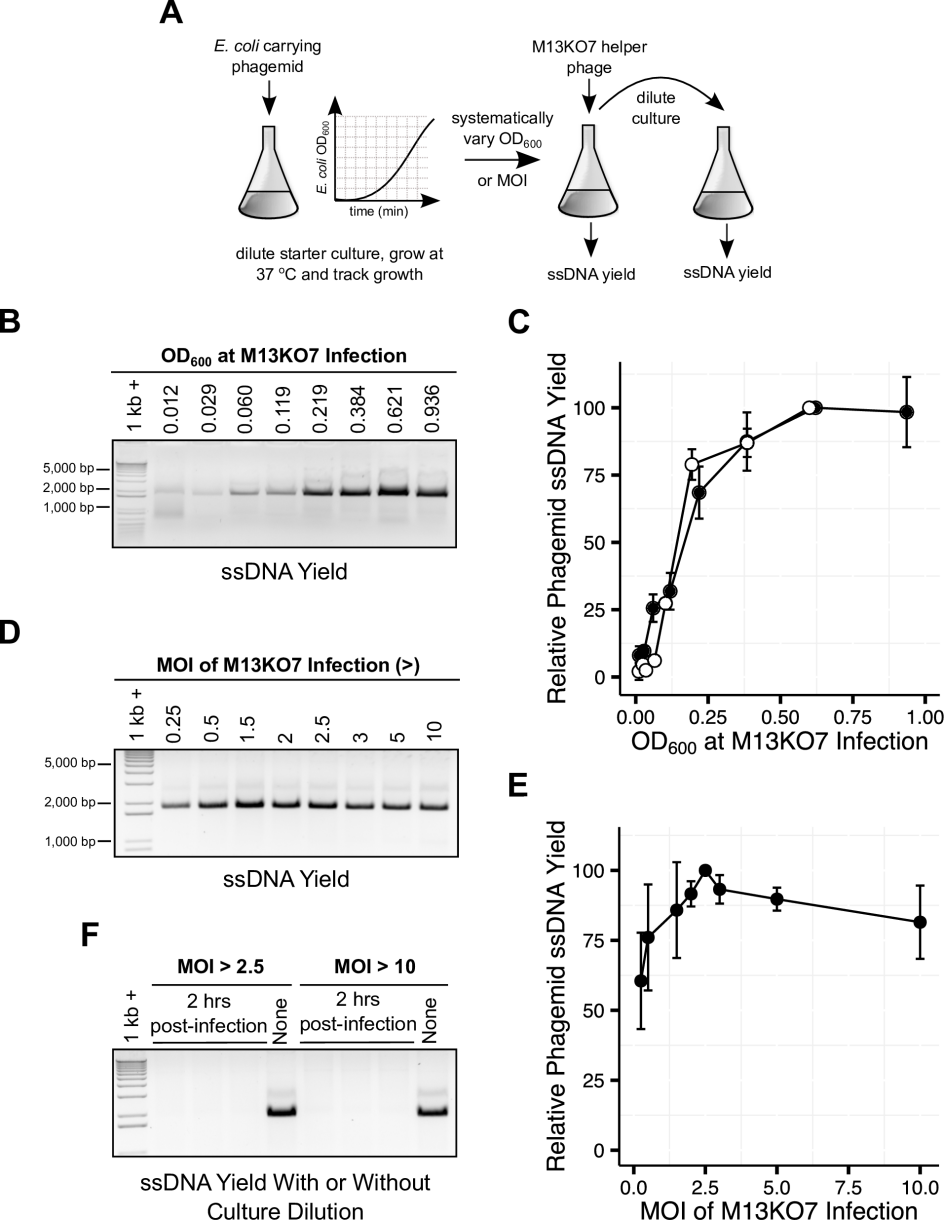
Optimization of the DH12S *E*. *coli* culture and phage infection conditions for phagemid ssDNA yield. (A) Schematic of the experimental design for the systematic analysis of the effect of culture OD_600_ at phage infection and multiplicity of infection (MOI) on ssDNA yield. Cultures were infected at varying OD_600_ or MOI and ssDNA yields were determined by proteinase K digestion of precipitated phage particles, followed by agarose gel electrophoresis. (B) OD_600_ at phage infection predicts ssDNA yield. (C) Increasing MOI beyond that which is necessary to infect all cells does not improve ssDNA yield. (D) Gel quantification of two biological replicates, each containing three technical replicates, of cultures infected at varying OD_600_ and identical MOI. (E) Quantification of ssDNA yields from cultures infected at varying MOI. Averages include two biological replicates, each containing three technical replicates. Error bars indicate the standard deviation. (F) Cultures that otherwise produce high yields of phagemid ssDNA, infected at MOI > 2.5 or 10, do not yield ssDNA when diluted 2 hours following phage-infection.

Mammalian transfection requires microgram quantities of DNA and thus high-yield, large-scale single-stranded phagemid production. However, one of the most significant obstacles to the reproducibility of phagemid production is that small variations in the experimental conditions can result in large differences in ssDNA yield. The growth medium, levels of aeration, multiplicity of infection (MOI) and growth stage of the target *E*. *coli* cells are all factors that could affect ssDNA yield. Different protocols provide different instructions for the timing of M13KO7 infection, including growing the cultures to early log-phase [21], for 3 hours [16], to an optical density at 600 nm (OD_600_) of 0.1 [17], or 0.05 [22]. Previous studies also recommend different MOIs, including 2–10 [21], 5 [17], or may not specify MOI [16]. In order to identify optimal infection conditions and reliable predictors of ssDNA yield, we performed systematic analysis of the culture and M13KO7 infection conditions. We infected DH12S *E*. *coli* with M13KO7 phage at an identical MOI (> 2.5) and varying cell densities as well as at identical densities but varying MOI (Fig 1A). We found that the DH12S *E*. *coli* culture density at the time of M13KO7 infection is a reliable predictor of ssDNA yield. ssDNA yield increases as OD_600_ increases, before plateauing at OD_600_ of approximately 0.6, yielding a several-fold increase compared to the ssDNA yields at OD_600_ of approximately 0.05 or 0.1 (Fig 1B and 1C). In cultures infected at the same OD_600_, we find that increasing the MOI does not increase yield (Fig 1D and 1E). Moreover, if cultures that otherwise produce high quantities of phagemid ssDNA are diluted before overnight incubation (Fig 1F) or after overnight incubation (S1 Fig), no ssDNA is produced. Thus, it appears that following the initial infection, the replication of M13KO7 may be insufficient for sustained phagemid production in the progeny of infected DH12S *E*. *coli* cells after multiple rounds of cell division. While the possibility that other factors may influence ssDNA yield in other *E*. *coli* strains cannot be excluded, the number of initially transduced DH12S cells is a reliable predictor of phagemid ssDNA yield.

**Fig 2:**
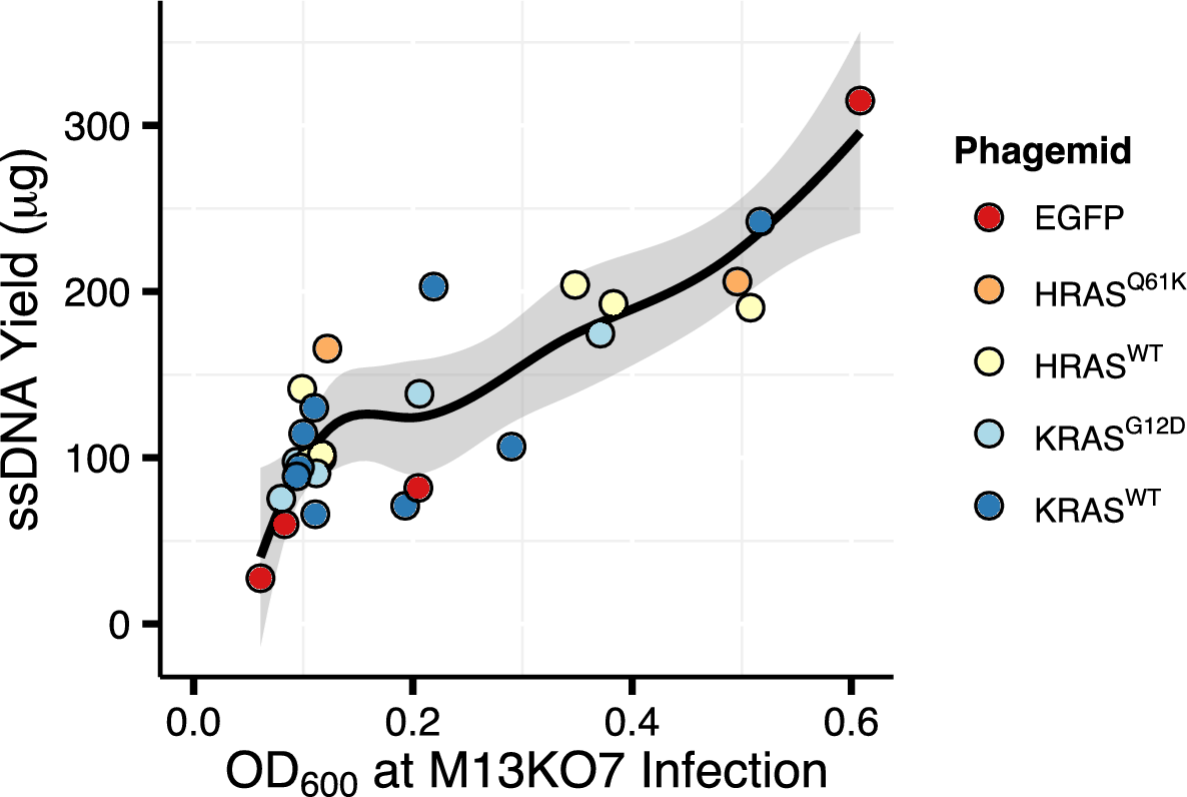
Culture density at time of helper phage infection predicts ssDNA yield. Scatter plot of large-scale (200 mL) ssDNA preparations infected at various MOI greater than one, following anion-exchange column purification. Black line represents the local polynomial regression (loess) curve and grey area the 95% confidence interval, as determined by R software.

Consistent with the systematic analysis of culture density at the time of infection, we also observed an increase in ssDNA yield with increasing OD_600_ at the time of M13KO7 infection in large-scale (200 mL) ssDNA preparations following anion-exchange column purification (Fig 2). By following these optimizations, it is possible to prepare more than 300 μg of highly pure phagemid ssDNA, with OD_260_/OD_280_ ratio of 1.83 ± 0.02 s.d. (n = 27), from a single 200 mL culture. If even larger quantities of ssDNA are desired, the number of cultures can be increased, since increasing the culture volume reduces the culture aeration and thus ssDNA yield. The ssDNA can be stored long-term at -20°C. We have successfully used over a year old ssDNA for second strand synthesis reactions.

Starting ssDNA yield and purity ultimately determine second strand synthesis product yield. In order to determine the yield of covalently closed double-stranded product, we used ssDNA purified by phenol:chloroform:isoamyl alcohol extraction (PCIA), silica spin columns, and anion-exchange columns for the second strand synthesis reaction. ssDNA purified only by PCIA did not yield any dsDNA product (data not shown). While both anion-exchange columns and silica spin columns yielded dsDNA product, that of the silica column-purified ssDNA contained mostly nicked and linear vector while anion-exchange column purified ssDNA yielded the highest quantities of covalently closed product. Hence, purification by anion-exchange columns results in ssDNA highly suitable for second strand synthesis.

### Purification of Highly Pure, Covalently-Closed, Double-Stranded Vectors

**Fig 3:**
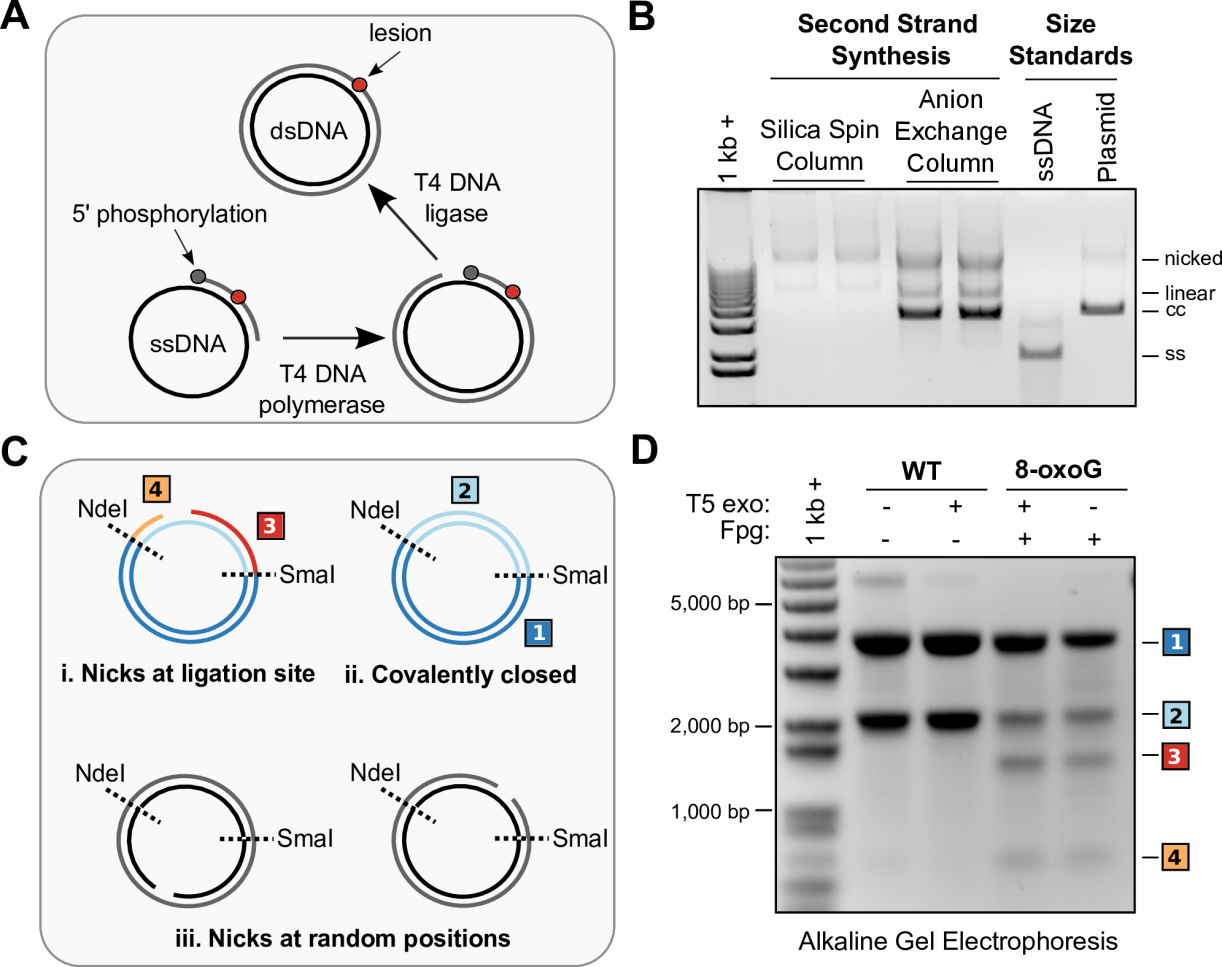
Optimizations for second strand synthesis. (A) Schematic of the second strand synthesis procedure. Synthetic 5’ phosphorylated ODNs containing the lesion of interest are annealed to phagemid single-stranded DNA, complimentary strands are synthesised by T4 DNA polymerase, and ligated by T4 DNA ligase. (B) Second strand synthesis of HRAS construct using ssDNA purified by silica spin columns or anion-exchange columns. ssDNA purified by anion-exchange column produces high yields of covalently closed product. (C) Schematic of the alkaline gel analysis of the construct nicks positions. Double-digest of pcDNA3.1(+)-HRAS with SmaI and NdeI produces two fragments (labelled 1 and 2). If the synthetic ODN that becomes part of the transcribed strand is not ligated, the transcribed strand fragment 2 produces two smaller fragments (3 and 4). (D) Alkaline gel analysis of HRAS constructs. Negative control HRAS^WT^ T5 exonuclease (T5 exo) treated, covalently closed construct produces only two bands and positive control Fpg nicked HRAS^8-oxoG^ constructs, treated and not treated with T5 exonuclease, produce the expected four bands. The anion-exchange purified HRAS^WT^ construct produces only two bands, indicating the nicks following second strand synthesis occur at random positions.

Several options exist for the purification of double-stranded products that can yield varying levels of DNA recovery, purity, adventitious background damage, such as oxidation or UV damage, as well as amount of covalently closed plasmid. Background DNA damage can confound mutagenesis studies and vector purity as well as the amount of covalently closed plasmid can significantly affect transfection efficiencies. The general strategy employed here for second strand synthesis and construct purification is depicted in Fig 3A and 3B (8-oxoguanine example). The second strand synthesis reactions do not result in all covalently closed product, but also contain the nicked and linear form of plasmid. The presence of nicked and linear product could be due to incomplete ligation by T4 DNA ligase, in which case the breaks would occur all in the same position and can be a confounding factor as single strand breaks can also be mutagenic, or at random positions. In order to determine whether the nicks occur all in the same position, we performed alkaline gel electrophoresis of constructs after digestion with restriction enzymes (SmaI and NdeI), selected such that each of these scenarios can be distinguished due to the different fragmentation patterns (Fig 3C and 3D, and S2 Fig) they would produce in denatured, single-stranded DNA. We find that while the positive control, Fpg nicked 8-oxoG construct, produces the expected lower molecular weight bands due to fragmentation of the nicked transcribed strand, the construct not treated with Fpg does not, indicating that nicks occur at random positions (Fig 3D), likely due to adventitious background single-strand break damage occurring during DNA manipulation procedures.

**Fig 4:**
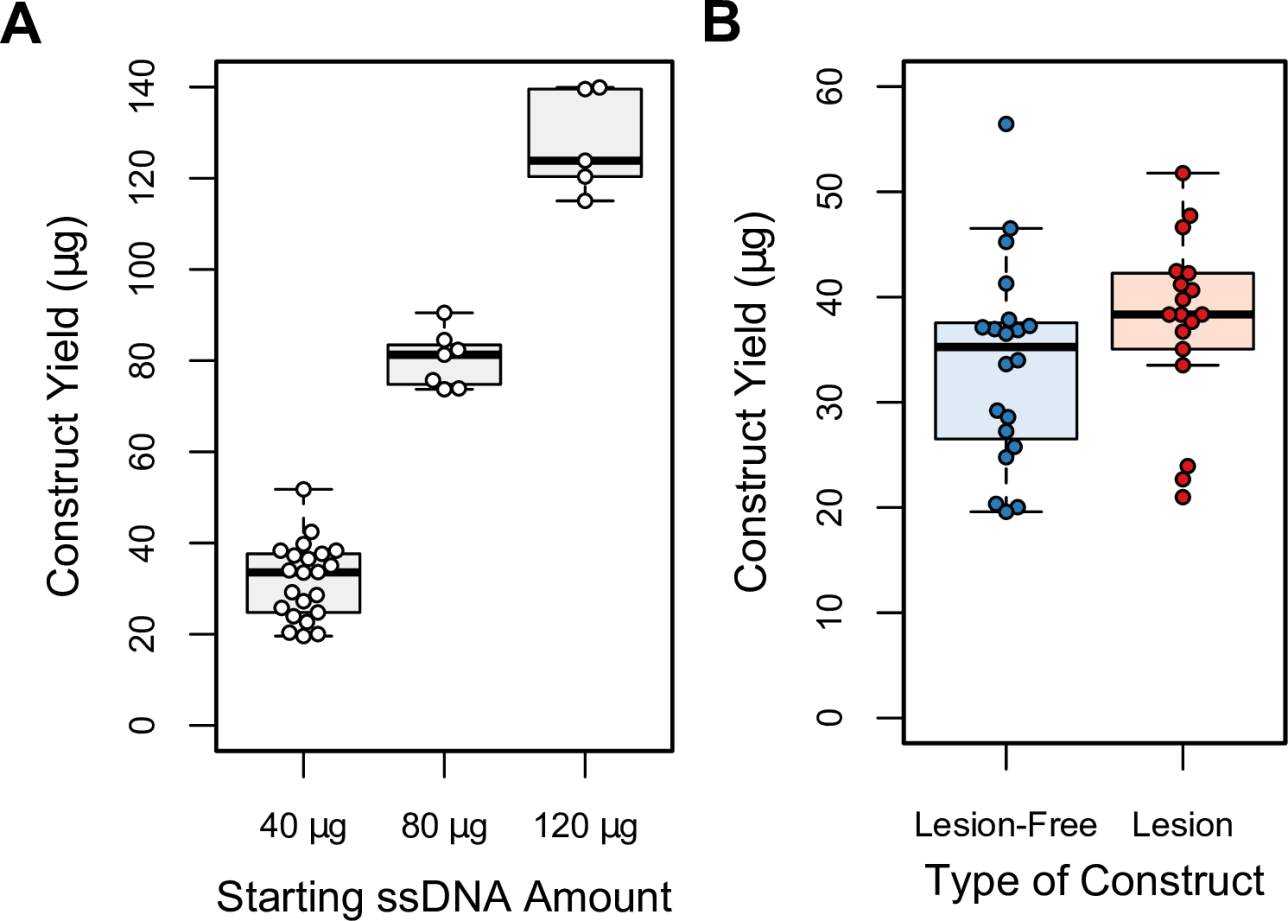
Second strand synthesis yields. (A) Box plots of second strand syntheses yields by starting amount of ssDNA indicate scalable and reliable yields. (B) Box plots comparing yields of lesion-free versus lesion-containing (8-oxoG, 5-OHU, or DHU) constructs, per 40 μg of ssDNA, or as expected per 40 μg of ssDNA if actual starting ssDNA amount was less (20–30 μg). Similar yields are obtained for lesion-free and lesion-containing constructs. Whiskers extend 1.5 times the interquartile range from the 25^th^ and 75^th^ percentiles, which are indicated by box limits. Center lines indicate the medians and circles represent the values for individual preparations. Determined by R software.

A previously used method, purification from low melting point agarose gels using β-agarase, allows for the isolation of initially closed circular product from the second strand synthesis reaction, but can result in significant levels of oxidation and single strand breaks (S3 Fig). Constructs purified by anion-exchange columns contain similar levels of nicked and linear form but produce significantly higher yield (36.1 μg ± 8.8 s.d., n = 38, versus about 15 μg [16] per 40 μg of starting ssDNA). Also, anion-exchange column purification can easily be scaled-up to hundreds of micrograms, without an increase in time or effort, by employing columns with larger capacity. We observe corresponding increases in product yields when scaling up second strand synthesis reactions (Fig 4A) and similar yields for lesion-free versus lesion-containing constructs (Fig 4B).

**Fig 5:**
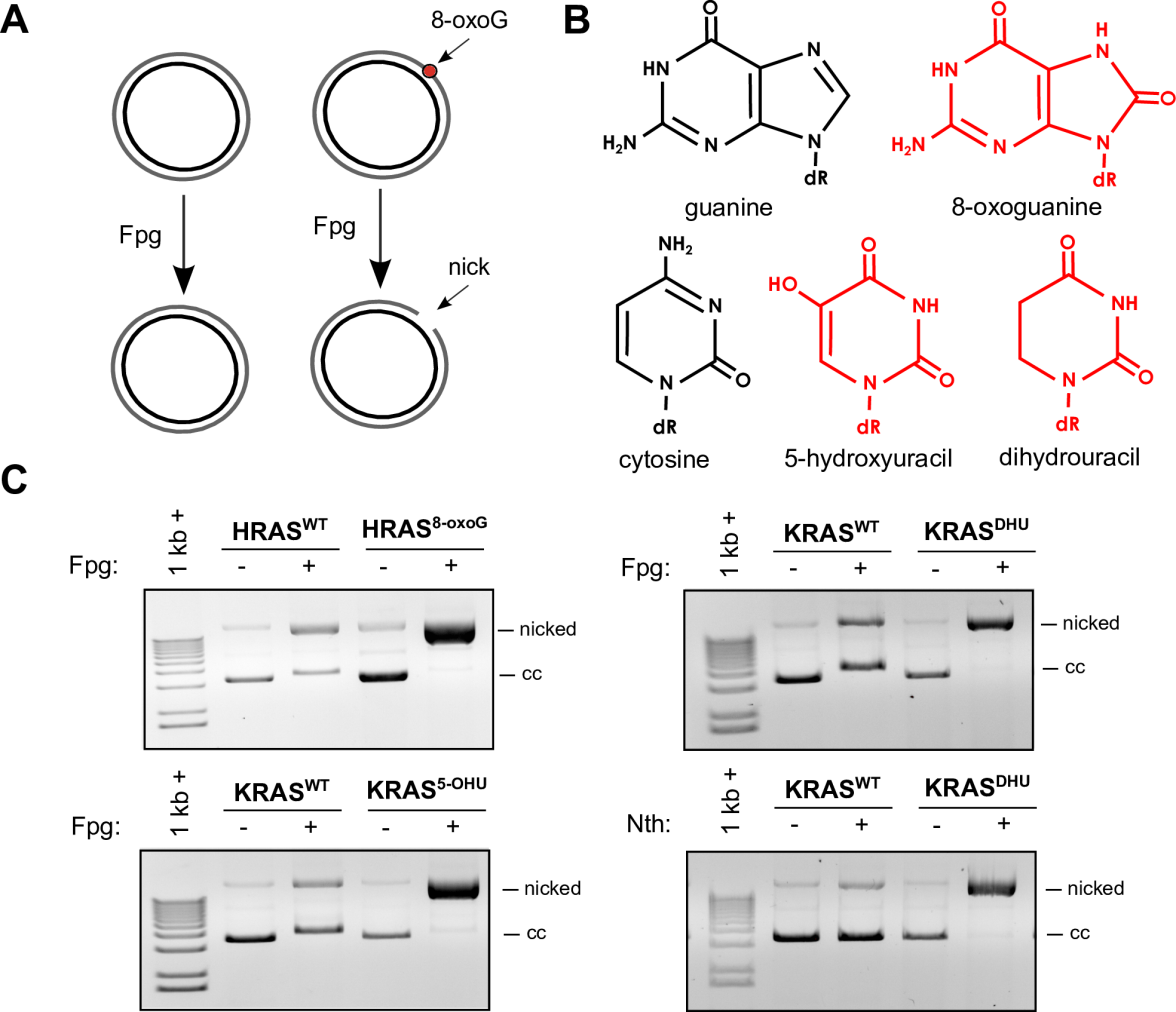
Lesion-containing construct quality controls. (A) Schematic of the Fpg nicking assay. Fpg cleaves damages, such as 8-oxoG and 5-OHU, leaving a single-strand break, converting the construct from covalently closed (cc) to nicked form. (B) Lesion structures. (C) Representative images of Fpg and Nth nicked T5 exonuclease-treated lesion-containing and lesion-free control constructs. Fpg cleaves 8-oxoG, 5-OHU, and DHU, nicking the lesion-containing constructs almost entirely, but not the lesion-free controls, and Nth cleaves DHU.

We performed second strand synthesis using ODNs containing three different oxidative lesions, including 8-oxoguanine, 5-hydroxyuracil (5-OHU), and dihydrouracil (DHU), and confirmed the presence of each lesion using the *E*. *coli* Formamidopyrimidine DNA glycosylase (Fpg) or Endonuclease III (Nth) nicking assays (Fig 5A and 5B). Fpg cleaves oxidative lesions such as 8-oxoG and 5-OHU [23], leaving a single strand break, in this case resulting in nicked plasmid that can be visualized on an agarose gel due to its altered migration pattern (Fig 5C). All constructs with second strands synthesized using the 8-oxoG or 5-OHU oligodeoxynucleotides were completely converted into the nicked form, indicating the presence of the lesions in these constructs as well as absence of detectable lesion-free dsDNA contamination, while those synthesized using lesion-free ODNs were not (Fig 5C). Dihydrouracil is a substrate for Nth [24], and the construct containing DHU was cut by Nth. While DHU has been described as a non-specific substrate for Fpg, DHU can be recognized by *B*. *stearothermophilus* Fpg in a manner similar to 8-oxoG [25], and can also be excised by *E*. *coli* Fpg from ODNs, albeit less efficiently than 8-oxoG from 8-oxoG:C pairs [26]. We find that the DHU-containing construct is completely converted into nicked form in the Fpg cleavage assay (Fig 5C), indicating that DHU is also a substrate of Fpg and Fpg is also a suitable enzyme for determining the presence of DHU.

**Fig 6:**
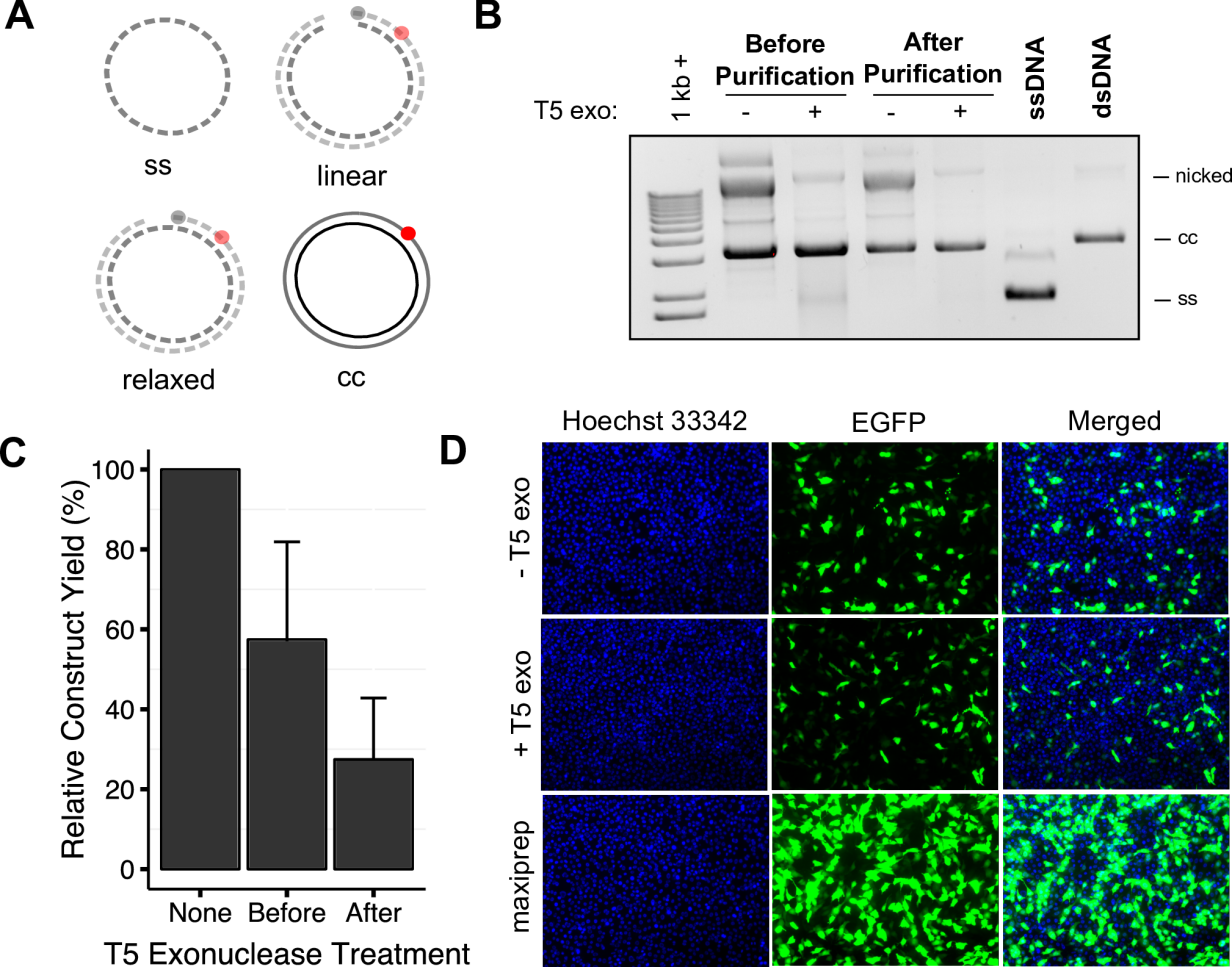
Optimization for DNA integrity and mammalian transfection. (A) Schematic representing T5 exonuclease digestion of nicked, linear, and ssDNA. (B) Representative gel electrophoresis of a construct with and without T5 exonuclease treatment prior to purification and after purification. (C) Construct yields after T5 exonuclease treatment after initial purification (after) or directly in the second strand synthesis reaction (before), relative to non-T5 exonuclease treated construct (none). Error bars represent the standard deviation. (D) Live cell images of Ogg1^-/-^ MEFs nucleofected with EGFP construct treated or not treated with T5 exonuclease or EGFP bacterial plasmid maxiprep, and stained with Hoechst 33342 dye. T5 exonuclease digestion of nicked and linear construct does not improve transfection efficiencies.

The presence of nicked and linear product could affect transfection efficiencies. In order to determine whether the presence of nicked vector affects transfection efficiency, we compared EGFP constructs purified using anion-exchange columns with or without enzymatic digestion of nicked, linear, and ssDNA using T5 exonuclease [15, 27], and EGFP bacterial maxiprep. T5 exonuclease treatment followed by anion-exchange column purification results in highly pure closed circular product (Fig 6A and 6B), albeit at the cost of reduction in yield. Higher yields can be obtained if the T5 exonuclease treatment is performed directly in the second strand synthesis reaction (Fig 6C). We find that treatment with T5 exonuclease does not improve transfection efficiencies, and EGFP constructs treated or not treated with the enzyme result in similar efficiencies (Fig 6D). We also found that bacterial EGFP plasmid purified using the same method and of identical purity results in higher efficiencies than both types of constructs, likely due to differences in the plasmid coiling.

### Lesion-Containing Constructs for the Study of the Phenotypic Consequences of Transcriptional Mutagenesis

8-oxoG can induce transcriptional mutagenesis and induce significant increases in extracellular-signal-regulated kinases 1 and 2 (ERK1/2) phosphorylation at 6 hours post-nucleofection in MEFs deficient in Ogg1, while it is almost immediately repaired in wild type (WT) cells [12]. Oncogenic mutant Ras can activate multiple downstream signaling cascades, including the Raf-MEK-ERK pathway, PI3K-AKT-mTOR, and RalGEF-Ral. and regulate a variety of cellular processes and cancer hallmarks, including cellular proliferation, survival, and angiogenesis [28]. However, downstream effectors of Ras in addition to ERK have not been previously studied in the context of TM. As the levels and duration of TM may vary, TM may differentially influence a variety of biological processes, each of which may have a different time course.

**Fig 7:**
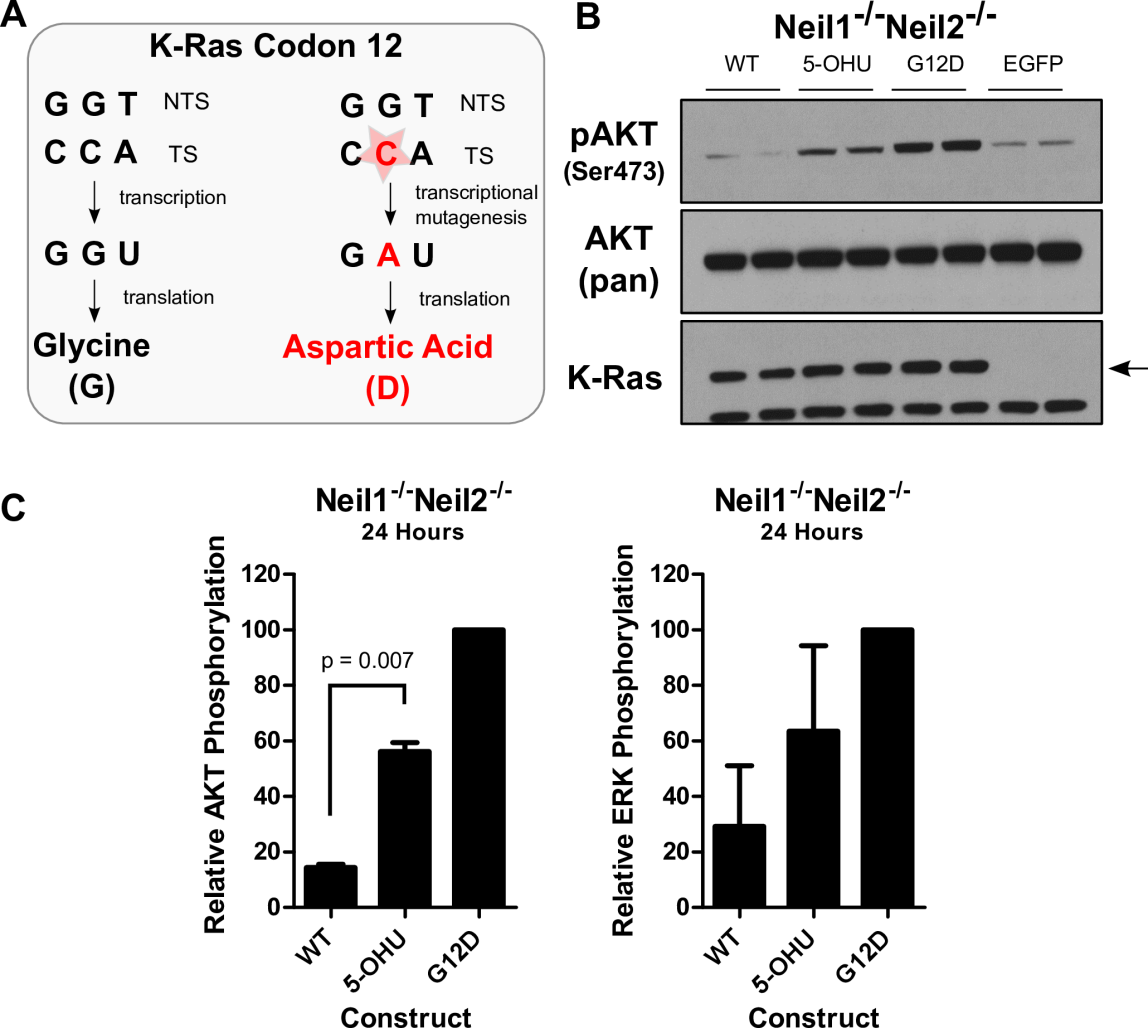
Phenotypic consequences of 5-hydroxyuracil in Neil1^-/-^Neil2^-/-^ MEFs. (A) Schematic of the construct, designed such that mutagenic bypass by RNA polymerase of 5-OHU produces the G12D mutant of K-Ras, leading to the activation of downstream oncogenic signaling. (B) Representative Western blot showing sustained increase in AKT phosphorylation at 24 hours post-nucleofection, with each sample loaded twice serving as a technical replicate. (C) Western blot quantification of two biological replicates, each containing two technical replicates, of pAKT and pERK relative to G12D positive control. The increase in AKT phosphorylation is statistically significant, as determined by a t-test. Error bars represent the standard error of the mean.

We predict that lesion transcriptional mutagenicity and persistence, due to repair deficiency, influence the robustness and longevity of signaling and its biological outcomes. Thus, we tested 5-OHU, which has been shown to be more highly transcriptionally mutagenic than other lesions such as 8-oxoG *in vitro*, for its ability to mediate oncogene activation *in vivo*. We placed 5-OHU in the G12 mutational hotspot of K-Ras, such that if TM occurs *in vivo*, similarly to *in vitro*, it would induce the production of oncogenic KRAS^G12D^ mutant transcripts and proteins, activating pathways downstream of Ras (Fig 7A). DNA glycosylases, including Neil1, Neil2, Neil3, and uracil-DNA glycosylase (UNG) have incision activity towards 5-OHU *in vitro* [29–32]. Thus, we employed cells doubly deficient in Neil1 and Neil2, Neil1^-/-^Neil2^-/-^ MEFs (Materials and Methods) in order to determine whether 5-OHU can induce TM and phenotypic change *in vivo*.

We observed increases in both AKT and ERK phosphorylation at 24 hours, demonstrating that TM-mediated signaling can last longer than previously known. These results also indicate that in cells with combined deficiency in Neil1 and Neil2, 5-OHU persists unrepaired long enough to induce TM and phenotypic change (Fig 7B and 7C), implicating Neil1 and/or Neil2 in the repair of 5-OHU *in vivo*. Moreover, TM can induce the activation of more than a single Ras effector pathway, which could have important implications *in vivo* due to the aberrant activation of more than a single cellular process. The increases in AKT phosphorylation are statistically significant, and while the increases in ERK phosphorylation are not, as both downstream pathways are dependent on mutant Ras, these phenotypic changes are likely to be biologically significant and may influence a variety of downstream biological consequences.

## Discussion

Here, we have presented a systematic analysis of factors influencing protocol reliability and yield of vectors containing site-specific base modifications in any position and sequence of interest. We further identified optimal conditions for reliable large-scale production of ultra-pure vectors highly suitable for applications in mammalian cell culture systems. We employed our improved protocol to study the phenotypic consequences of 5-OHU in cells deficient in both Neil1 and Neil2 DNA glycosylases and found that TM can induce sustained oncogenic signaling and activate more than one downstream effectors of Ras. It is likely that such sustained TM-mediated oncogene activation of multiple pathways downstream of Ras is sufficient for and can lead to phenotypic consequences beyond biochemical signaling such as induction of proliferation, acquisition of a permanent DNA mutation and oncogenic transformation via retromutagenesis, or increases in DNA damage [7], activation of the DNA damage response and oncogene-induced senescence [33]. Deficiency in both Neil1 and Neil2 is sufficient to allow the occurrence of 5-OHU-mediated TM *in vivo*, implicating Neil1 and/or Neil2 in the repair of 5-OHU *in vivo*. However, the relative contributions of Neil1 and Neil2, as well as other enzymes known to have activity towards 5-OHU *in vitro*, in the repair of 5-OHU remain to be determined.

This streamlined protocol should prove useful for the study of the mutagenicity, physiological consequences, and repair of individual lesions in a variety of contexts in basic and translational research, including emerging areas that have not yet been thoroughly investigated. For example, while a great proportion of the physiologically diverse cells in mammals exist in a non-proliferative state, very little is known how DNA damage present in non-dividing cells, such as quiescent stem cells, pre-malignant senescent cells, or terminally differentiated neurons and glia, contributes to the aging process or the development and pathology of cancer, neurodegenerative disease or other illnesses associated with DNA damage. Since reporters of transcriptional mutagenesis do not require DNA replication, they constitute a tool suitable for the study of DNA damage not only in dividing but also non-dividing cells. Due to the method’s versatility and ability to position defined lesions in any sequence and reporter of interest, some of its potential applications in translational research include high-content screening of anti-cancer compounds targeting DNA repair as well as monitoring therapeutic responses in cultured patient tumour samples. While our focus has been mammalian systems, it is important to note that the applications of this technique are not limited to only mammalian cells but also, after an appropriate choice of vector, other systems such as bacteria. Also, while the vectors we have used are non-replicating in the absence of the SV40 large T antigen, in order to avoid the confounding factor of replicative mutagenesis, as opposed to transcriptional mutagenesis, transfection into cell lines that contain it or other episomally replicating vectors could be used to study replicative mutagenesis.

This and other similar methods rely on prior knowledge of the modified base, its stability in experimental procedures, and its successful chemical synthesis into an ODN. A variety of oxidative base lesions (e.g. 8-oxoguanine, 5-hydroxyuracil, dihydrouracil, thymine glycol, spiroiminohydantion), alkylating DNA damage lesions (e.g. O6-methylguanine), those produced by reactive nitrogen species, UV, chemotherapeutic drugs (e.g. cisplatin) or other DNA damaging compounds (e.g. aflatoxin) have already been successfully incorporated into ODNs. Future advances in endogenous DNA damage detection and characterization methods and nucleic acid synthetic chemistry will likely further expand the growing number DNA damage lesions that can be studied using this and similar techniques. A further improvement of the present system would be the development of efficient and reliable technologies for the targeted introduction of DNA damage lesions into genomic DNA.

## Supporting Information

S1 Fig ssDNA yields of diluted cultures. ssDNA yields determined by proteinase K digestion of precipitated phage, followed by gel electrophoresis, from infected *E*. *Coli* cells 2 hours post-infection, after overnight incubation, and after dilution of the first overnight culture and a second overnight incubation. High yields of ssDNA are only present in undiluted cultures after an overnight incubation.
(TIF)

S2 Fig Determination of the second strand synthesis product nicks positions. (A) pcDNA3.1(+)-HRAS plasmid map generated using Angular Plasmid (http://angularplasmid.vixis.com/) and sequence surrounding the 8-oxoG lesion and ligation site for second strand synthesis. (B) Overexposure of the alkaline gel electrophoresis. (C) The same samples separated on non-denaturing agarose gel in TBE buffer.
(TIF)

S3 Fig Gel purification of constructs. Low melting point agarose (LMP) and β-agarase purification of constructs. Covalently closed forms of KRAS^5-OHU^ and EGFP maxiprep were purified from SeaPlaque GTG LMP agarose using β-agarase (Lonza) as per the manufacturer’s instruction and digested with Fpg as described in Materials and Methods. LMP purification can result in nicking and high levels of oxidation.
(TIF)

S4 Fig M13KO7 preparation. DH12S *E*. *coli* not containing phagemid were infected with M13KO7 phage as per the same protocol for phagemid production and ssDNA was purified using PCIA extraction. M13KO7 HRAS^8-oxoG^ second strand synthesis reaction, HRAS^WT^ ssDNA, HRAS^WT^ plasmid maxiprep and M13KO7 ssDNA preparation were resolved on an agarose gel to compare sizes. The faint upper band in the ssDNA preparation has the same migration pattern as M13KO7 ssDNA. While we do not observe significant M13KO7 ssDNA contamination in purified constructs not treated with T5 exonuclease (Fig 6B), treatment with T5 exonuclease can be employed if minimizing ssDNA contamination is preferred.
(TIF)

S1 Table ODN sequences. Sequences of oligodeoxynucleotides containing 5’ phosphorylation (P), 8-oxoguanine (8-oxoG), 5-hydroxyuracil (5-OHU), or dihydrouracil (DHU), used for second strand synthesis.
(TIF)

S2 Table Supplementary list of buffers and media. Composition of commercial buffers used and buffers and media prepared.
(TIF)
