# Supplementary figures and images for: Efficient and Reliable Production of Vectors for the Study of the Repair, Mutagenesis, and Phenotypic Consequences of Defined DNA Damage Lesions in Mammalian Cells
Source: PLoS One. 2016 Jun 30;11(6):e0158581. doi: 10.1371/journal.pone.0158581 (PMC4928824; doi:10.1371/journal.pone.0158581)

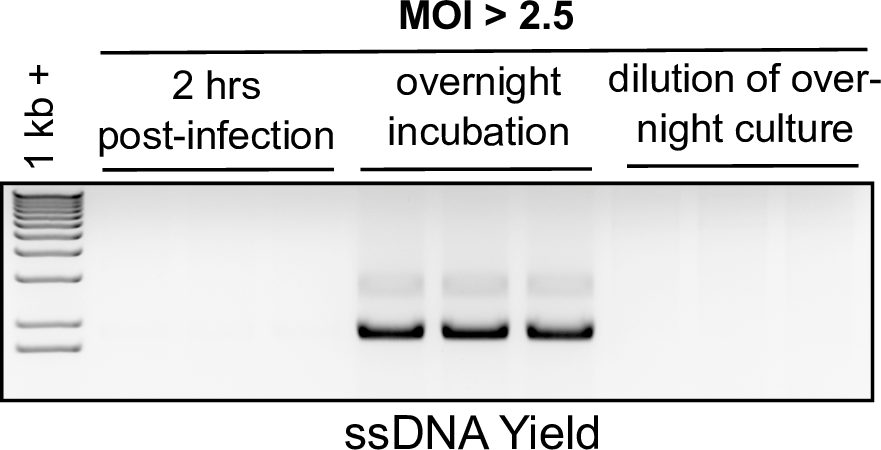

Supplement: S1 Fig — ssDNA yields determined by proteinase K digestion of precipitated phage, followed by gel electrophoresis, from infected E. Coli cells 2 hours post-infection, after overnight incubation, and after dilution of the first overnight culture and a second overnight incubation. High yields of ssDNA are only present in undiluted cultures after an overnight incubation. (TIF) [file pone.0158581.s001.tif]

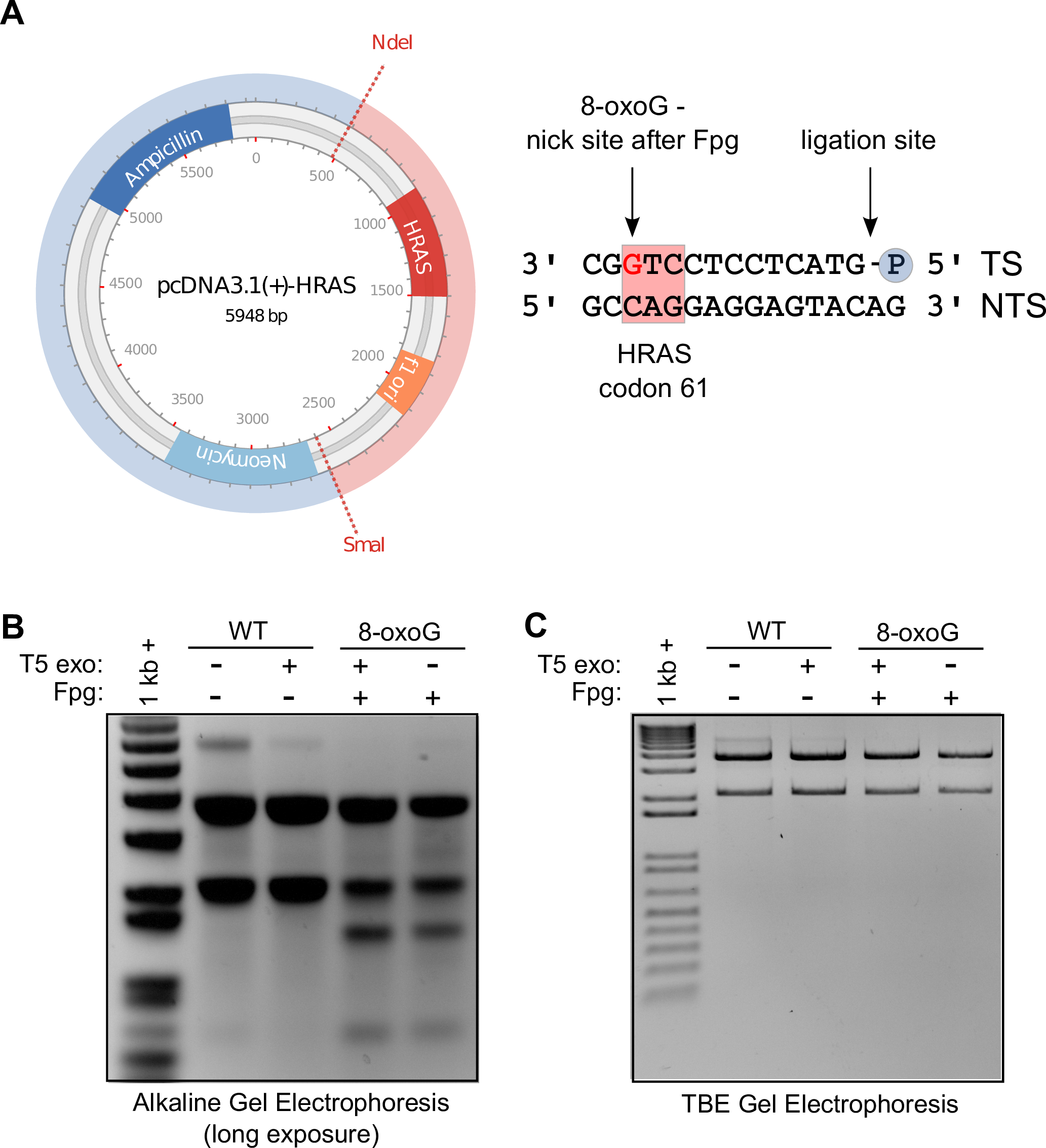

Supplement: S2 Fig — (A) pcDNA3.1(+)-HRAS plasmid map generated using Angular Plasmid (http://angularplasmid.vixis.com/) and sequence surrounding the 8-oxoG lesion and ligation site for second strand synthesis. (B) Overexposure of the alkaline gel electrophoresis. (C) The same samples separated on non-denaturing agarose gel in TBE buffer. (TIF) [file pone.0158581.s002.tif]

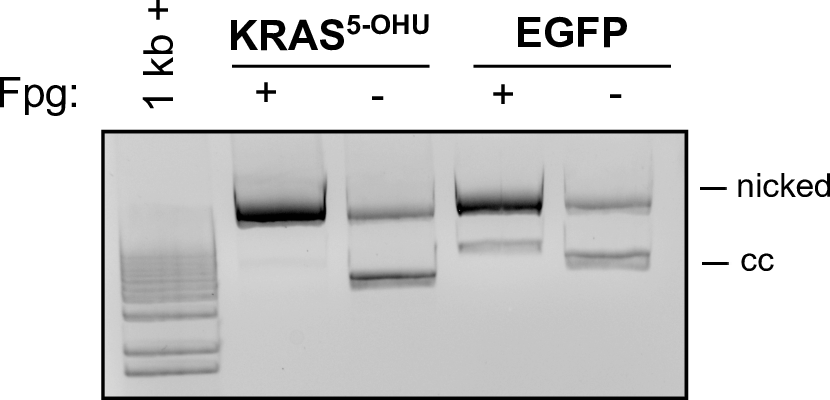

Supplement: S3 Fig — Low melting point agarose (LMP) and β-agarase purification of constructs. Covalently closed forms of KRAS5-OHU and EGFP maxiprep were purified from SeaPlaque GTG LMP agarose using β-agarase (Lonza) as per the manufacturer’s instruction and digested with Fpg as described in Materials and Methods. LMP purification can result in nicking and high levels of oxidation. (TIF) [file pone.0158581.s003.tif]

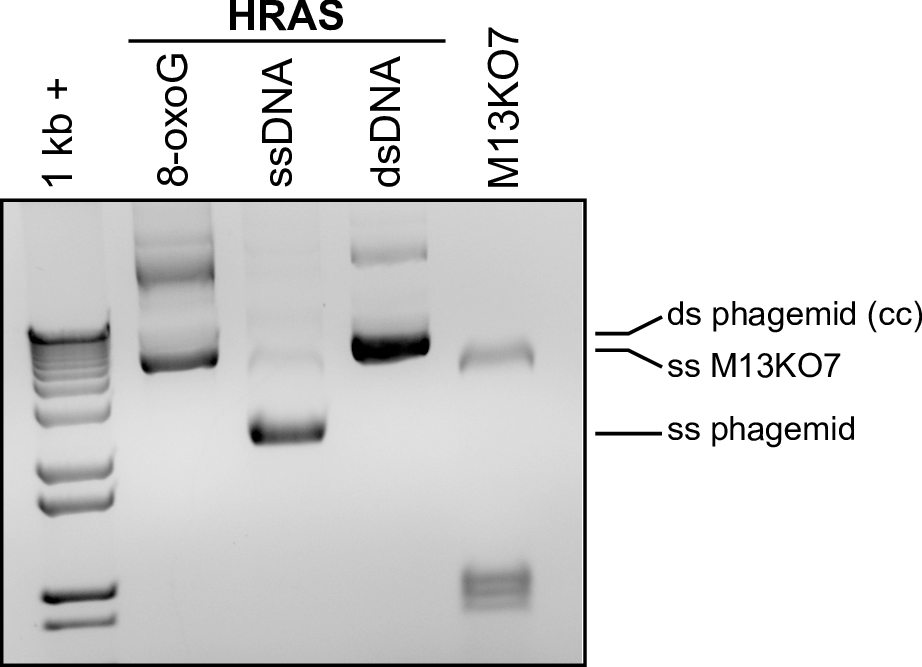

Supplement: S4 Fig — DH12S E. coli not containing phagemid were infected with M13KO7 phage as per the same protocol for phagemid production and ssDNA was purified using PCIA extraction. M13KO7 HRAS8-oxoG second strand synthesis reaction, HRASWT ssDNA, HRASWT plasmid maxiprep and M13KO7 ssDNA preparation were resolved on an agarose gel to compare sizes. The faint upper band in the ssDNA preparation has the same migration pattern as M13KO7 ssDNA. While we do not observe significant M13KO7 ssDNA contamination in purified constructs not treated with T5 exonuclease (Fig 6B), treatment with T5 exonuclease can be employed if minimizing ssDNA contamination is preferred. (TIF) [file pone.0158581.s004.tif]

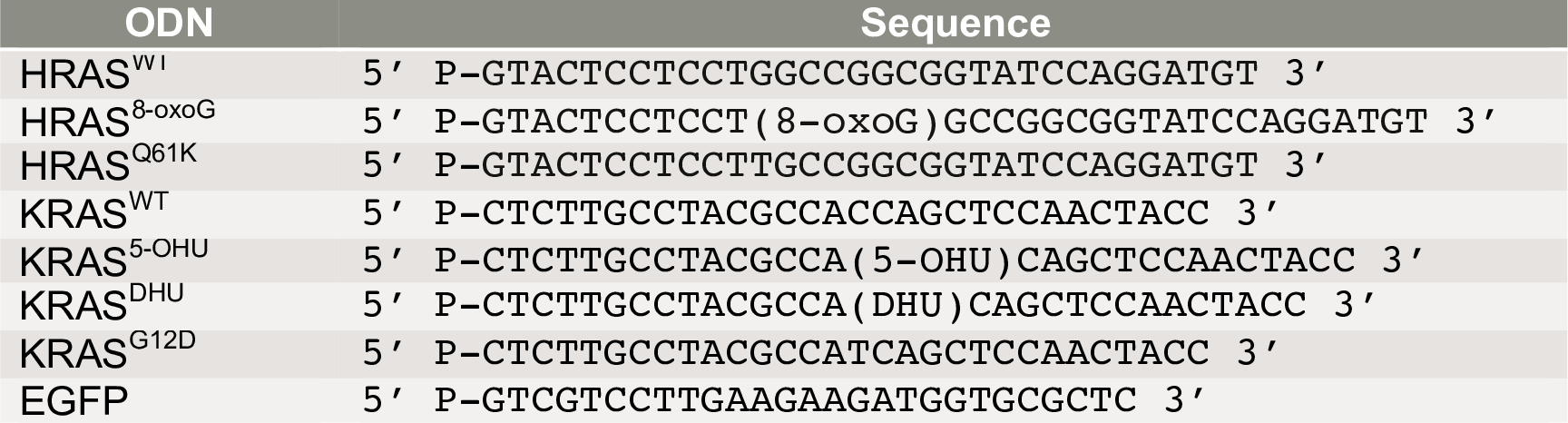

Supplement: S1 Table — Sequences of oligodeoxynucleotides containing 5’ phosphorylation (P), 8-oxoguanine (8-oxoG), 5-hydroxyuracil (5-OHU), or dihydrouracil (DHU), used for second strand synthesis. (TIF) [file pone.0158581.s005.tif]

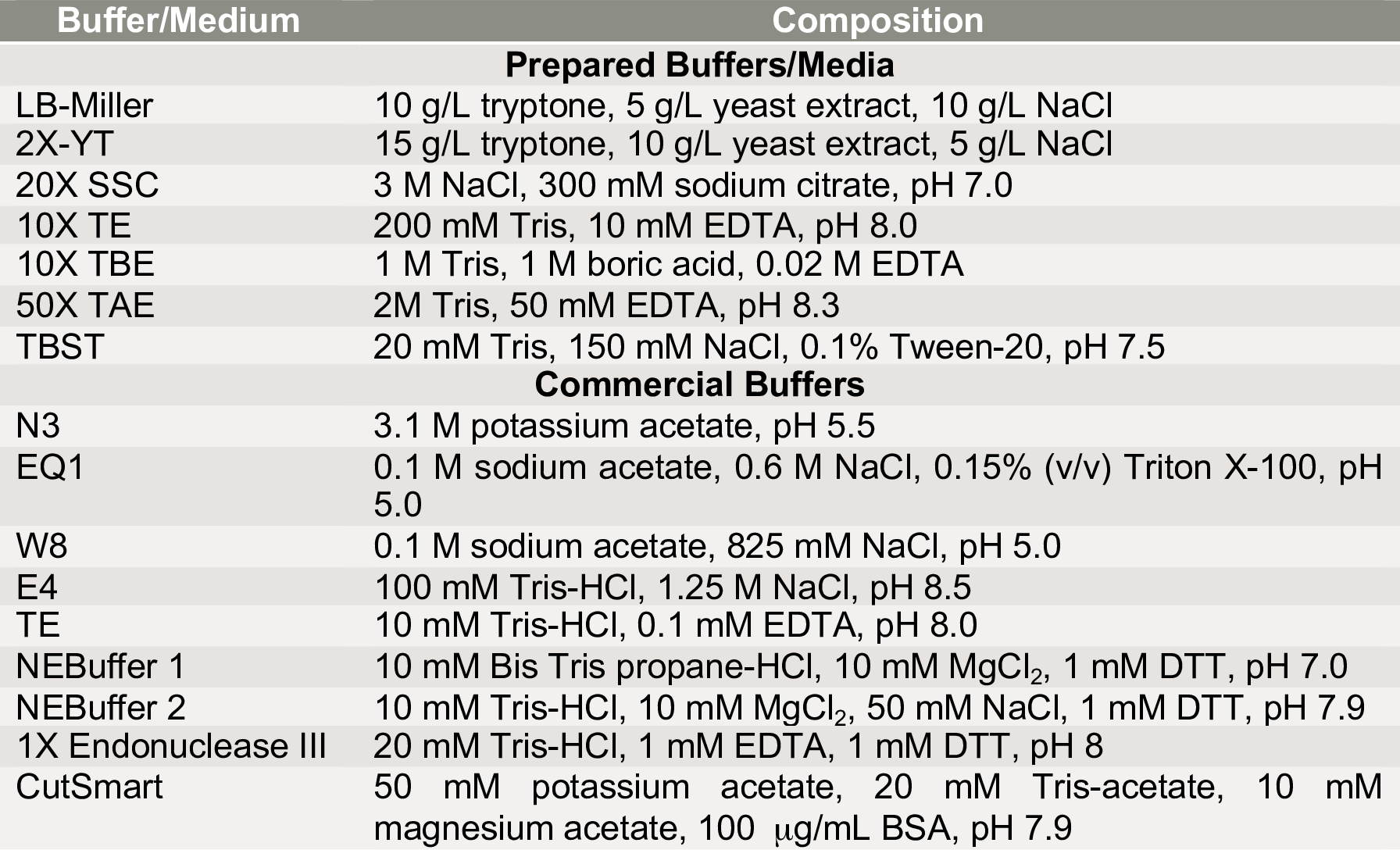

Supplement: S2 Table — Composition of commercial buffers used and buffers and media prepared. (TIF) [file pone.0158581.s006.tif]
